# Klinefelter Syndrome in Childhood: Variability in Clinical and Molecular Findings

**DOI:** 10.4274/jcrpe.5121

**Published:** 2018-05-18

**Authors:** Neşe Akcan, Şükran Poyrazoğlu, Firdevs Baş, Rüveyde Bundak, Feyza Darendeliler

**Affiliations:** 1 Near East University Faculty of Medicine, Department of Pediatric Endocrinology, Nicosia, Cyprus; 2 İstanbul University İstanbul Faculty of Medicine, Department of Pediatric Endocrinology, İstanbul, Turkey; 3 University of Kyrenia Faculty of Medicine, Department of Pediatric Endocrinology, Kyrenia, Cyprus

**Keywords:** Ambigious genitalia, cryptorchidism, disorders of sex development, speech impairment

## Abstract

**Objective:**

Klinefelter syndrome (KS) is the most common (1/500–1/1000) chromosomal disorder in males, but only 10% of cases are identified in childhood. This study aimed to review the data of children with KS to assess the age and presenting symptoms for diagnosis, clinical and laboratory findings, together with the presence of comorbidities.

**Methods:**

Twenty-three KS patients were analyzed retrospectively. Age at admission, presenting symptoms, comorbid problems, height, weight, pubertal status, biochemical findings, hormone profiles, bone mineral density and karyotype were evaluated. Molecular analysis was also conducted in patients with ambiguous genitalia.

**Results:**

The median age of patients at presentation was 3.0 (0.04-16.3) years. Most of the cases were diagnosed prenatally (n=15, 65.2%). Other reasons for admission were scrotal hypospadias (n=3, 14.3%), undescended testis (n=2, 9.5%), short stature (n=1, 4.8%), isolated micropenis (n=1, 4.8%) and a speech disorder (n=1, 4.8%). The most frequent clinical findings were neurocognitive disorders, speech impairment, social and behavioral problems and undescended testes. All except two patients were prepubertal at admission. Most of the patients (n=20, 86.9%) showed the classic 47,XXY karyotype. Steroid 5 alpha-reductase 2 gene and androgen receptor gene mutations were detected in two of the three cases with genital ambiguity.

**Conclusion:**

Given the large number of underdiagnosed KS patients before adolescence, pediatricians need to be aware of the phenotypic variability of KS in childhood. Genetic analysis in KS patients may reveal mutations associated with other forms of disorders of sex development besides KS.

## What is already known on this topic?

Klinefelter syndrome (KS) is the most common chromosomal disorder in humans but patients with KS are often diagnosed late in life. Less than 10% of patients are diagnosed before puberty.

## What this study adds?

Neurodevelopmental, psychological and verbal disorders with undescended testes are the findings of Klinefelter syndrome (KS) in childhood that should alert the physician. Further molecular analyses should also be considered in KS patients with ambiguous genitalia to exclude additional gene mutations.

## Introduction

Klinefelter syndrome (KS) is the most common (1/500–1/1000) chromosomal disorder in humans (1). Men with KS are often diagnosed late in life, usually during investigation for infertility and the mean age of diagnosis is commonly in the mid-30s (1,2). The diagnosis rate of KS is estimated to be only 25% (2,3). Furthermore, less than 10% of KS patients are diagnosed before puberty (4).

Although no firm diagnosis guidelines for KS exist and extreme heterogeneity in clinical and genetic presentation is found (5), the usual key findings in KS are primary testicular failure with small testes, hypergonadotrophic hypogonadism, tall stature with eunuchoid body proportions, neurocognitive impairment mainly related to language processing disability and varying degrees of social, behavioral, and learning difficulties (3,5). Gynecomastia, metabolic syndrome, osteoporosis, cryptorchidism, decreased penile size, psychiatric disturbances, inguinal hernia, mitral valve prolapse, growth hormone deficiency, hypothyroidism, hypoparathyroidism and increased risk of autoimmune diseases are some of the other reported abnormalities associated with KS (2,6). Newborns with KS generally present with a normal male phenotype, although ambiguous genitalia has been reported to be associated with KS (3,4). Phenotypic variation may depend on the severity of the expression of genetic defects, androgen deficiencies, androgen receptor (AR) sensitivities (i.e., CAG repeats polymorphism), or randomly skewed inactivation of the additional X chromosome material (4).

Patients who are unaware that they have KS are assumed to be relatively healthy and do not require treatment. Because of this, clinical description of the more mildly affected patients is not generally available leading to a limited account of the full spectrum of KS phenotypes (1). Given the insufficient awareness of this syndrome and the typical delay in diagnosis until after puberty, our aim was to review the data of our pediatric patients with KS to evaluate the age and reason for diagnosis, as well as the clinical and laboratory findings and presence of comorbid conditions.

## Methods

A retrospective medical chart review was performed to collect data from the pediatric endocrinology outpatient clinics of İstanbul University, İstanbul, Turkey and Near East University Nicosia, Northern Cyprus, between January 1992 and February 2017. The definition of KS, as an inclusion criterion for this study, required the availability of a karyotype consisting of an X chromosome polysomy and at least one Y chromosome, either as a single lineage or as a mosaicism (7). Twenty-three patients with a confirmed diagnosis of KS and sufficient documented data were included in the study. Age at the time of diagnosis, chief complaint at admission and presenting symptoms, parents’ ages during pregnancy and existing comorbidities were evaluated. Physical examination findings, pubertal status and secondary sex characteristics, presence of gonadal failure and gynecomastia were investigated. Clinical and laboratory parameters, including height, weight, body mass index (BMI), testicular volumes, fasting blood glucose levels, fasting insulin levels, homeostatic model assessment–insulin resistance (HOMA-IR), total testosterone, luteinizing hormone (LH), follicle-stimulating hormone (FSH), thyroid hormones, *25*-*hydroxyvitamin D [*25(OH)D_3_*], bone mineral density (BMD) and* different karyotype disorders of KS were recorded.

The standard deviation score (SDS) for height, weight and BMI were calculated according to reported data for healthy Turkish boys (8). To compare the data, the study group was divided into three age groups (<3, 3-9, >9 years) to determine the age-specific velocity in growth (3). A hologic anthropomorphic lumbar spine scan analysis was used and sex-specific z-scores for BMD were derived from the analysis. When BMD z-scores are between −1.0 and −1.9, “at risk for low *BMD* or bone mineral content for chronologic age” is used for terminology, and when BMD z-scores are less than or equal to −2.0, the terms “low *BMD*” or “low bone mineral content for chronologic age” were preferred instead of “osteoporosis” (9). 25(OH)D_3_ levels were compared in cases with low and normal BMD values. The diagnosis of a neurocognitive disorder and the need for special education were retrieved from the medical records. Molecular analysis of the steroid 5 alpha-reductase 2 (*SRD5A2*) and *AR* genes was performed in three cases which presented with ambiguous genitalia. Patient records and information were anonymized and de-identified before analysis.

### Statistical Analysis

Statistical Package for Social Sciences Software (SPSS 21, Chicago, IL, USA) was used for the analysis. All continuous variables were expressed as the median, minimum and maximum values. Age, height, weight, BMI and hormone levels were shown as median values. The Kruskal-Wallis test was used for the comparisons of height, weight and BMI SDS among the three age groups. Categorical variables were expressed as numbers and percentages. The Mann-Whitney U test was used to compare 25(OH)D_3_ levels. A p value <0.05 was considered significant.

## Results

### Demographics and Clinical Characteristics

The median age of the patients at presentation was 3.0 (0.04-16.30) years. Most of the cases were diagnosed prenatally by amniocentesis because of advanced parental age. These prenatally diagnosed patients had no complaints at admission, except for two patients whose age at presentation was 3 years and whose additional complaints were short stature and undescended testis, respectively. The median maternal age and paternal age during pregnancy among all cases were 40.0 (22.0-46.0) and 38.5 (24.0-52.0) years, respectively. The details of the chief complaints on admission and the findings during the initial physical examination are shown in Table 1. Patients with speech disorder and amylogenesis imperfecta were diagnosed during genetic investigation for their main complaints in the department of physiotherapy and rehabilitation and pediatric dentistry. In the patient with short stature, all tests including growth hormone stimulation tests were found to be normal. 

Most of the cases were prepubertal at admission. The pubertal patients (n=2) presented with complaints of undescended testes or micropenis. Regarding the follow-up data of all patients, the early signs of pubertal development were observed in three of the prepubertal patients and in total we observed pubertal progress in five patients during follow-up. In three of the five pubertal patients, testicular volumes had ceased to increase, pubertal testes remained small without additional enlargement, testicular failure had occurred and testosterone replacement therapy was started. The other two pubertal patients were in very early puberty with testicular volumes of 4 mL and they need more time to observe their pubertal progress (Table 1). Only two of the prepubertal patients had atrophic/hypoplasic testes (testis volume <0.5 mL) at admission. 

Undescended testes were found in almost one-third of all patients during the initial physical examination, but only two of them presented with this complaint. The cases with scrotal hypospadias or gynecomastia are also described in Table 1. One of the patients with scrotal hypospadias was raised with a female identity. This patient underwent bilateral laparoscopic gonadectomy and sex steroid therapy was initiated to stimulate pubertal changes in accordance with a female identity. Neurocognitive disorders and the need for special education were determined in almost half of the patients. Severe hypotonia and neuromotor retardation were observed in only one case.

Median height, weight and BMI SDS in KS patients younger than three years, between three and nine years, and after nine years are shown in Table 2. Despite the increase in growth status, which was observed clinically, the difference was not statistically significant.

### Laboratory and Radiological Findings

At the time of minipuberty hormone levels showed variability. In the prepubertal period, LH, FSH and total testosterone levels were all in prepubertal ranges. During the pubertal period, the median testosterone level was found to be within normal ranges when the median LH and FSH levels began to increase, which indicated gonadal failure (Table 3).

Thyroid hormone levels and *thyroid antibodies *were normal in all patients. Hyperinsulinemia was observed in two patients (one prepubertal and one pubertal) who had BMI values >2 SDS, although they had normal glucose tolerance. HOMA-IR results were 4.9 and 5.8 in these patients, respectively.

The L1-L4 vertebral BMD z-score was evaluated in 13 patients. The median (range) level of the BMD z-score was −1.0 (−3.6 to 0.7). Two pubertal patients had low *BMD* (z-score −3.2 and −3.6) and five patients (one pubertal and four prepubertal) were at risk for low *BMD* (z-score: −1.2, −1.3, −1.4, −1.4, and −1.8). The median 25(OH)D_3_* of the whole cohort was 19.1 *ng/mL (6.6-41.3). Median 25(OH)D_3_* levels were 13 *ng/mL (8.2-41.3) and 18.3 ng/mL (6.6-19.3) in the cases who had a BMD z score ≤−1 and a BMD z score >−1. No statistically significant difference was found between these groups in terms of BMD z score.

### Karyotype and Molecular Analyses

Most of the patients (n=20, 86.9%) showed the classic 47,XXY karyotype (Table 4). All three of the cases presenting with ambiguous genitalia had the 47,XXY karyotype. One of these three cases was homozygous for the p.G196S mutation in exon 4 of the *SRD5A2* gene, and the other was heterozygous for the p.P892L mutation in exon 8 of the *AR* gene. However, no mutation was detected in either *AR* or *SRD5A2* in the third KS patient with scrotal hypospadias (Table 4). Both of the mutations detected have been previously described in the Human Genome Mutation Database.

## Discussion

KS is the most common sex chromosome aneuploidy in live male births, but less than 10% of cases are identified before puberty (10). This finding is worrying because these cases will present with complex comorbidities, such as hypogonadism, osteopenia/osteoporosis, metabolic syndrome, neurodevelopmental and psychosocial dysfunction all of which will adversely affect quality of life (3). With an early diagnosis, the complications of these comorbidities during follow-up can be minimized (11). According to our data, most of our patients were referred because of their prenatal diagnosis. Prenatal or early diagnosis can provide early and close monitoring of potential comorbidities (3). Early treatment of cryptorchidism, speech therapy with social training, close monitoring for learning disabilities and psychological support to both patients and parents are benefits which may be expected from early diagnosis. A special focus on nutrition and exercise for both bone mineralization and metabolic syndrome after the age of three years may be beneficial as has been suggested in the literature (3). In addition to the prenatal diagnosed patients, our series also includes a limited number of cases diagnosed in childhood and adolescence. The low ratio of cases diagnosed at pediatric ages leads to a concern about pediatric cases being missed. For this reason, increasing the rate of early diagnosis in childhood would be of immense benefit.

Speech disability may be the only sign during infancy (5). Disability in language processing, which requires additional educational help, was detected in almost half of our cases, but speech disorder as a primary complaint was present in only one single case. Language difficulties have been identified in 70%-80% of children with KS, starting at an early age (6,12). As language and learning disabilities become manifest during infancy, clinicians should bear in mind a possible diagnosis of KS in infants showing these disabilities. Health providers who deal with speech disorders should be informed about KS to prevent delay in diagnosis. It has been suggested that genital anomalies, such as micropenis, undescended testis and hypospadias, are rarely present at birth (5). Undescended testis was observed in almost one-third of the patients (n=8) in our case series, and half of them (n=4) also had a speech disorder. The almost 10-fold higher prevalence of KS in cryptorchid boys supports the indication for a karyotype analysis in these children (13). Cryptorchidism and mild developmental disorders may be warning signs for KS. Therefore, giving karyotype priority to children with undescended testes, particularly those who have accompanying speech impairment, is recommended.

Growth velocity is known to be accelerated by the age of three years with a modest increase in adolescence (3). Even though the statistical significance was not meaningful, the median height SDS increased after the age of three and seemed to increase again after puberty. Therefore, KS should also be considered in the differential diagnosis of boys whose height SDS increases after three years of age and increases further in puberty although this would be a delayed diagnosis. In our cohort, only one case, who had also a prenatal diagnosis, presented with short stature when he was aged three years. There are a few reported cases of KS with short stature, secondary to growth hormone deficiency (14,15). However, we did not detect growth hormone deficiency in this patient.

As traditionally described, patients with KS have small testes. The progressive increase in testes volume does not occur during puberty, both testes remaining small and firm (5). The course of puberty was observed in five patients in our case series, and three of them also had available adulthood records in the follow-up data. Their records showed that testicular volumes showed no increase and remained small without additional enlargement. Therefore, although pubertal findings may be initially observable there is a risk of pubertal arrest, and a close follow-up during puberty is needed to begin hormonal replacement therapy at the right time.

Some of the earliest studies on minipuberty in infants with KS indicated that these boys could already have presented with biochemical signs of hypergonadotrophic hypogonadism. Conversely, recent large studies reported normal concentrations of testosterone and normal LH levels in minipuberty despite the finding that total testosterone concentrations were below the median of the total testosterone levels of the control group (3,16,17). In the current study, hormone levels showed variability, which was difficult to interpret, at the time of minipuberty.

Some clinical conditions associated with KS, such as diabetes and metabolic syndrome, worsen progressively with advancing age (5). IR and metabolic syndrome were reported in 24% and 7% of KS children of ages 4-12 years (3,18). We detected hyperinsulinemia in 8.7% (n=2) of the patients. However, the rate of hyperinsulinemia would probably increase over time during the follow-up of remaining patients.

Low BMD is prevalent in patients with KS (5,19,20). This rate is 30.4% (n=7) in our case series. The presence of osteopenia or osteoporosis in KS children may not manifest until puberty (19). However, KS patients have been reported to have an impaired bone mineral status that begins early in life (20). Four of the seven KS patients who had BMD SDS <−l were also in the prepubertal period in the current study.

The 25(OH)D_3_ levels were previously reported to be significantly lower in KS patients than in controls (20). In our case series, nearly half of the patients had 25(OH)D_3_ levels lower than 20 ng/mL. However, no significant difference was found in median 25(OH)D_3_ levels between cases with normal BMD and those with BMD <−1. Except for two cases, all patients with BMD <−1 had 25(OH)D_3_ levels lower than 20 ng/mL. The etiopathogenesis of impaired bone mineral status in KS patients may be multifactorial, including KS-specific bone characteristics and/or low testosterone levels. Poor vitamin D levels may also contribute to impaired *BMD* in children with KS.

About 80%-90% of KS cases have 47,XXY karyotype, and the remaining cases may have a mosaic karyotype (46,XY/47,XXY), additional X or Y chromosomes (48,XXXY or 48,XXYY), or structurally abnormal X chromosomes (e.g., 47,X,iXq,Y) (1). Mosaicism (mainly 46,XY/47,XXY) is present in 10%-20% of KS patients (5). Interestingly, we did not find any 46,XY/47,XXY mosaicism, although we had 48,XXYY (n=1), 48,XXXY (n=1), and 47,XXY/48,XXYY (n=1) karyotypes. Men with mosaic KS may be more androgenized, with larger testicular volumes and better hormonal profiles, than their non-mosaic counterparts (21). In the present case series, we did not have patients with 46,XY/47,XXY mosaicism. Some 46,XY/47,XXY cases could have been overlooked because of the silent phenotype. Therefore, the silent phenotype or minor findings of mosaic KS may result in the underdetection of KS children, a problem which pediatricians should be more aware of.

New disorders of sexual development (DSDs) nomenclature includes common entities such as Turner syndrome and KS under the title of sex chromosome DSDs (22,23). However, KS classically has complete male sex differentiation and ambiguous genitalia are generally not recognized as associated features of KS (23,24,25). The current study included three cases of ambiguous genitalia with KS. There are three cases of 47,XXY karyotype and *AR* gene mutation published in the literature (26,27,28). Two of them were reported to have complete androgen insensitivity syndrome whereas the other one had partial androgen insensitivity syndrome. Our case with *AR* gene mutation had partial androgen insensitivity syndrome, with heterozygous p.P892L mutation in exon 8. Partial androgen insensitivity syndrome developed in this case, probably because of the presence of the heterozygous AR mutation concurrent with random X inactivation of the healthy allele. It has been reported that the variation in phenotype could be explained by hormonal and genetic background differences, including androgen receptor polymorphism in the CAG_n_ repeat and skewed inactivation of additional genetic material on the X chromosome in KS patients (1,2,4,29). The one patient with homozygous *SRD5A2* gene mutation was considered a coincidence because of the consanguinity of his parents. The last patient with genital ambiguity had no detectable mutation in either *AR* or *SRD5A2* genes. This case could have been genetically investigated further for other genes associated with DSD [e.g., sex-determining region Y (*SRY*), dosage-sensitive sex reversal (*DSS*) or *DAX-1 *gene locus on the X chromosome] that could cause genital anomalies. From our perspective, we suggest that analyzing some gene mutations, especially *AR* and *SRD5A2* genes, in KS cases with ambiguous genitalia would be useful. The evaluation of some gene mutations in KS cases with ambiguous genitalia is essential to provide accurate genetic counseling for other members of the family. Moreover, explaining the linkage between KS and ambiguous genitalia, by excluding the other gene mutations that cause genital ambiguity, may be possible through this evaluation.

### Study Limitations

Our study has several limitations. First, due to the nature of the study, we had to rely on data from medical records. Secondly, the lipid profile and serum levels of other reproductive hormones (e.g., serum estradiol, inhibin B, anti-Mullerian hormone and INSL3) were not examined. Thirdly, the small sample size limited us from obtaining statistically significant results. These shortcomings can be overcome in future prospective studies with samples of larger size.

## Conclusion

Our data indicate that KS remains largely underdiagnosed in childhood. Pediatricians need to be aware of the phenotypic variability of KS. Specifically, neurodevelopmental, psychological and verbal disorders with undescended testes in childhood should prompt clinicians to evaluate the child in terms of KS. Further molecular analyses should be considered in KS patients with ambiguous genitalia to provide comprehensive genetic counseling to the family. Indeed, early diagnosis is essential to address age-specific challenges with timely treatment and rehabilitation to minimize the problems that patients with XXY face with and to mitigate some of the complications seen in late diagnosed cases.

## Figures and Tables

**Table 1:**
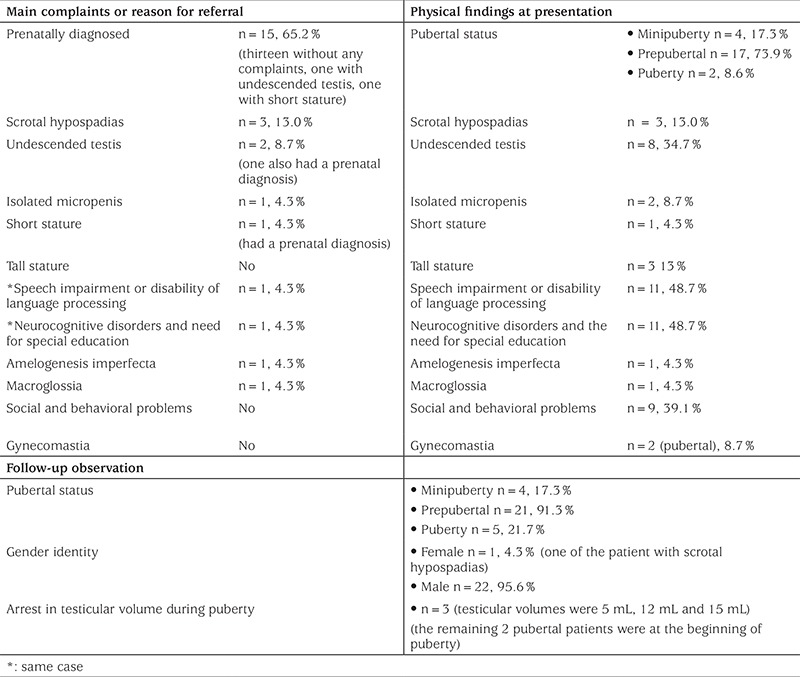
Complaints and physical findings of Klinefelter syndrome patients at presentation and follow-up observation

**Table 2:**
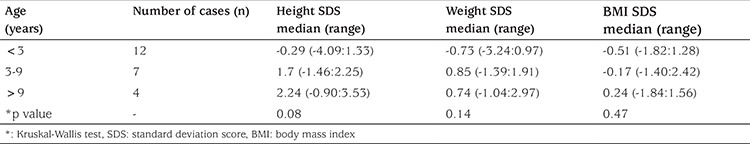
Height, weight and body mass index standard deviation score in Klinefelter syndrome patients by age at admission

**Table 3:**
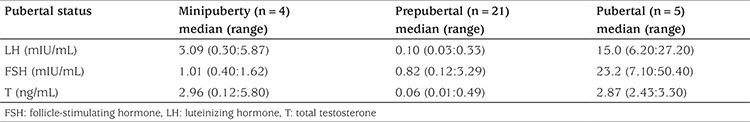
Hypothalamic-pituitary-gonadal axis hormone levels by pubertal status

**Table 4:**
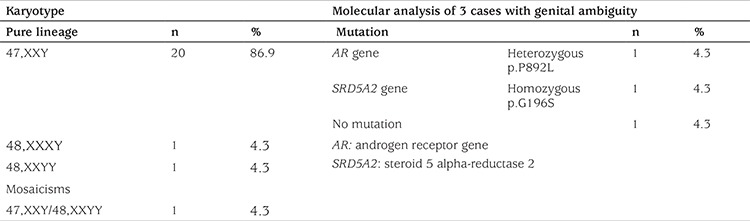
Karyotypes of Klinefelter syndrome patients and molecular analysis of three cases with genital ambiguity
